# Introduction to Special NIST Journal of Research Issue on Additive Manufacturing

**DOI:** 10.6028/jres.119.014

**Published:** 2014-10-10

**Affiliations:** 1 Beginning in late August John Slotwinski will be affiliated with the Johns Hopkins University Applied Physics Laboratory

Additive manufacturing (AM) or “3D-printing,” which is another term often used to describe all additive manufacturing processes but is in fact just a subset of AM technologies, has received substantial and rapidly growing attention in recent years in both the popular press and in technical publications. Phrases like “make anything at any time,” “art to part,” and “complexity is free,” along with notions of printing high-stress aerospace components, or fully-functioning cars or robots, all in one process, contribute to today’s AM fervor. Recent niche successes in biomedical and aerospace applications offer a possible glimpse of future things to come in AM. Taken as a whole the vision for AM is imaginative, impressive, and inspiring, and it may even be achievable someday.

However, the full vision of AM is not realized today due to technical hurdles that prevent more widespread dissemination of AM technologies. Recent road-mapping efforts^2^,^3^ have captured the most significant of these technical hurdles, including a lack of understanding of AM materials, a desire for in-process sensing and control, and the need for methods for qualifying AM materials, processes, and parts.

As guest editor for this special issue, it is my pleasure to assemble this snapshot of the metals-based additive manufacturing work that takes place in NIST’s Engineering Laboratory. This work addresses the needs identified by industry, and is infrastructural in nature, which means that the measurements and standards that are developed in this work are intended to benefit all of industry, and ultimately help propagate these technologies both nationally and internationally. The work is very interdisciplinary, a common characteristic of successful AM research, and leverages diverse talent from across NIST.

In *Mechanical Properties of Austenitic Stainless Steel Made by Additive Manufacturing*, Bill Luecke and John Slotwinski examine the mechanical properties of a stainless steel material that is commonly used in additive manufacturing, including the effects of build orientation and post-processing heat treatments, and compare it to traditionally made stainless steel. In *Sustainability Characterization for Additive Manufacturing*, Mahesh Mani, Kevin Lyons, and S. K. Gupta examine the potential environmental impacts of AM, and propose a methodology for sustainability characterization of AM. Shawn Moylan *et al.* describe an indirect method for measuring AM machine performance and capabilities in *An Additive Manufacturing Test Artifact*, including the design rationale for the proposed test artifact, a review of previous AM test artifacts, and a demonstration of how the proposed test artifact can be used to characterize and improve an AM process. In *Characterization of Metal Powders Used for Additive Manufacturing* Slotwinski *et al.* employ various methods to characterize the properties of the input metal powders used in some AM processes, and apply those methods to both characterize the consistency of nominally identical powder and to demonstrate how powder that is recycled multiple time in an AM process changes. Finally, in *Porosity Measurements and Analysis for Metal Additive Manufacturing Process Control*, Slotwinski *et al.* use ultrasonic non-destructive evaluation methods for characterizing the porosity of parts made via additive manufacturing, discuss the insertion of an ultrasonic sensor for process monitoring in an AM system, and proposed a design for such process monitoring.

I hope that you find this special issue interesting and informative. Please feel free to contact the individual authors with comments or questions.

## Footnotes

2 Measurement Science Roadmap for Metal-Based Additive Manufacturing, http://events.energetics.com/NIST-AdditiveMfgWorkshop/pdfs/NISTAdd_Mfg_Report_FINAL.pdf, May 2013.

3 D. L. Bourell *et al.*, eds., Roadmap for Additive Manufacturing – Identifying the Future of Freeform Processing, 2009.

